# Five-year follow-up of a phase I trial of donor-derived modified immune cell infusion in kidney transplantation

**DOI:** 10.3389/fimmu.2023.1089664

**Published:** 2023-07-11

**Authors:** Matthias Schaier, Christian Morath, Lei Wang, Christian Kleist, Gerhard Opelz, Thuong Hien Tran, Sabine Scherer, Lien Pham, Naruemol Ekpoom, Caner Süsal, Gerald Ponath, Florian Kälble, Claudius Speer, Louise Benning, Christian Nusshag, Christoph F. Mahler, Luiza Pego da Silva, Claudia Sommerer, Angela Hückelhoven-Krauss, David Czock, Arianeb Mehrabi, Constantin Schwab, Rüdiger Waldherr, Paul Schnitzler, Uta Merle, Vedat Schwenger, Markus Krautter, Stephan Kemmner, Michael Fischereder, Manfred Stangl, Ingeborg A. Hauser, Anna-Isabelle Kälsch, Bernhard K. Krämer, Georg A. Böhmig, Carsten Müller-Tidow, Jochen Reiser, Martin Zeier, Michael Schmitt, Peter Terness, Anita Schmitt, Volker Daniel

**Affiliations:** ^1^ Department of Nephrology, Heidelberg University Hospital, Heidelberg, ;Germany; ^2^ TolerogenixX GmbH, Heidelberg, ;Germany; ^3^ German Center for Infection Research, German Center for Infection Research (DZIF), Thematic Translational Unit (TTU)-Infections of the Immunocompromised Host (IICH), Partner Site Heidelberg, Heidelberg, ;Germany; ^4^ Department of Hematology, Oncology and Rheumatology, Heidelberg University Hospital, Heidelberg, ;Germany; ^5^ Institute of Immunology, Heidelberg University Hospital, Heidelberg, ;Germany; ^6^ Department of Nuclear Medicine, Heidelberg University Hospital, Heidelberg, ;Germany; ^7^ Transplant Immunology Research Center of Excellence, Koç University, Istanbul, ;Türkiye; ^8^ Department of Clinical Pharmacology and Pharmacoepidemiology, Heidelberg University Hospital, Heidelberg, ;Germany; ^9^ Department of General, Visceral and Transplantation Surgery, Heidelberg University Hospital, Heidelberg, ;Germany; ^10^ Institute of Pathology, Heidelberg University Hospital, Heidelberg, ;Germany; ^11^ Center for Infectious Diseases, Virology, Heidelberg University Hospital, Heidelberg, ;Germany; ^12^ Department of Gastroenterology, Heidelberg University Hospital, Heidelberg, ;Germany; ^13^ Department of Nephrology, Klinikum der Landeshauptstadt Stuttgart, Stuttgart, ;Germany; ^14^ Transplant Center, University Hospital Munich, Ludwig-Maximilians University (LMU), Munich, ;Germany; ^15^ Division of Nephrology, Department of Internal Medicine IV, University Hospital Munich, Ludwig-Maximilians-Universität München (LMU), Munich, ;Germany; ^16^ Department of General, Visceral, and Transplant Surgery, University Hospital Munich, Ludwig-Maximilians-Universität München (LMU), Munich, ;Germany; ^17^ Medical Clinic III, Department of Nephrology, University Hospital Frankfurt, Goethe University, Frankfurt am Main, ;Germany; ^18^ Fifth Department of Medicine (Nephrology/Endocrinology/Rheumatology/Pneumology), University Medical Centre Mannheim, University of Heidelberg, Mannheim, ;Germany; ^19^ Division of Nephrology and Dialysis, Department of Medicine III, Medical University of Vienna, Vienna, ;Austria; ^20^ Department of Medicine, Rush University, Chicago, IL, ;United States

**Keywords:** transplantation - kidney, tolerance, cell therapy, regulatory B (Breg) cells, phase I (drug development)

## Abstract

**Background:**

The administration of modified immune cells (MIC) before kidney transplantation led to specific immunosuppression against the allogeneic donor and a significant increase in regulatory B lymphocytes. We wondered how this approach affected the continued clinical course of these patients.

**Methods:**

Ten patients from a phase I clinical trial who had received MIC infusions prior to kidney transplantation were retrospectively compared to 15 matched standard-risk recipients. Follow-up was until year five after surgery.

**Results:**

The 10 MIC patients had an excellent clinical course with stable kidney graft function, no donor-specific human leukocyte antigen antibodies (DSA) or acute rejections, and no opportunistic infections. In comparison, a retrospectively matched control group receiving standard immunosuppressive therapy had a higher frequency of DSA (log rank *P* = 0.046) and more opportunistic infections (log rank *P* = 0.033). Importantly, MIC patients, and in particular the four patients who had received the highest cell number 7 days before surgery and received low immunosuppression during follow-up, continued to show a lack of anti-donor T lymphocyte reactivity *in vitro* and high CD19^+^CD24^hi^CD38^hi^ transitional and CD19^+^CD24^hi^CD27^+^ memory B lymphocytes until year five after surgery.

**Conclusions:**

MIC infusions together with reduced conventional immunosuppression were associated with good graft function during five years of follow-up, no *de novo* DSA development and no opportunistic infections. In the future, MIC infusions might contribute to graft protection while reducing the side effects of immunosuppressive therapy. However, this approach needs further validation in direct comparison with prospective controls.

**Trial registration:**

https://clinicaltrials.gov/, identifier NCT02560220 (for the TOL-1 Study). EudraCT Number: 2014-002086-30.

## Introduction

The long-term survival of kidney grafts continues to be hampered by two reasons. First, conventional immunosuppressive therapy is still unable to prevent long-term kidney graft loss, and a significant proportion of patients lose their grafts due to chronic (antibody-mediated) rejection (1). Second, immunosuppressive drugs have significant side effects such as infections and cancers that lead to the death of transplanted patients with functioning allografts (2). For this reason, one of the main goals in the field of solid organ transplantation is to create durable graft acceptance without immunosuppressive therapy, i.e., tolerance. Tolerance can be achieved by inducing durable chimerism in the setting of combined kidney and bone marrow transplantation (3). However, this approach is associated with the side effects of conditioning therapy and carries the risk of graft-versus-host disease (4). For example, in a Northwestern University trial on the induction of mixed chimerism using FCRx therapy in 37 HLA-mismatched living-donor kidney transplant recipients, two patients suffered graft-versus-host disease, two patients experienced graft loss and a total of up to 3 patients ultimately died (4, 5). The subsequent FREEDOM-1 and FREEDOM-2 clinical trials, using the same therapy, were recently terminated prematurely presumably for the same reasons (6, 7). Rather than transplanting the entire immune system, there have been recent efforts to modify the recipient’s immune system through cellular therapies to accept the transplanted organ (8–12). In the recently published The One study, patients receiving different cellular therapies, e.g., regulatory T lymphocytes and tolerogenic dendritic cells, along with reduced conventional immunosuppressive therapy were compared with a reference group of patients receiving regular immunosuppressive therapy after living donor kidney transplantation (10). This study demonstrated the safety and feasibility of regulatory T lymphocyte therapies together with tacrolimus alone in preventing kidney graft rejection while reducing infectious complications (11, 12). Currently, efforts are underway to further improve the efficacy of such an approach by introducing a chimeric antigen receptor (CAR) into regulatory T lymphocytes that recognizes HLA-A2 on transplanted organs. In this way, regulatory T lymphocytes accumulate in an HLA-A2 positive graft when introduced into an HLA-A2 negative recipient (13).

We have previously published the results of a phase I trial using modified donor peripheral blood mononuclear cells (MIC) together with reduced standard immunosuppressive therapy to prevent rejection in 10 living donor kidney transplant recipients (14, 15). Four of the 10 patients who had received the highest cell dose of 1.5 × 10^8^ MIC per kg body weight 7 days prior to surgery showed donor-specific immunosuppression, as evidenced by a lack of cellular stimulatory reactivity after transplantation when tested against their respective donors *in vitro*. These patients also had higher numbers of IL-10-producing CD19^+^CD24^hi^CD38^hi^ transitional B lymphocytes as well as of other regulatory B lymphocyte subsets at various stages of maturation which are thought to have regulatory properties. The T-cell response against the donor was increased after depletion of B lymphocytes *in vitro*, suggesting that regulatory B lymphocytes were pathophysiologically relevant to establish donor-specific unresponsiveness. These results were also supported by a gene expression signature consistent with the *COMBINED-g7* consensus signature of operational tolerance in three of the four patients, suggesting that we actively induced an operationally tolerant phenotype by high-dose MIC treatment one week before surgery (14, 15). In the current analysis, we wanted to investigate how this cell therapy approach together with reduced immunosuppressive therapy affected the continued clinical course of treated patients. We compared the clinical outcomes of the 10 MIC patients to outcomes of 15 matched transplanted controls. In addition, we provide immunological outcomes of MIC patients up to year 5 after surgery.

## Patients and methods

### Patients

**Figure 1:**
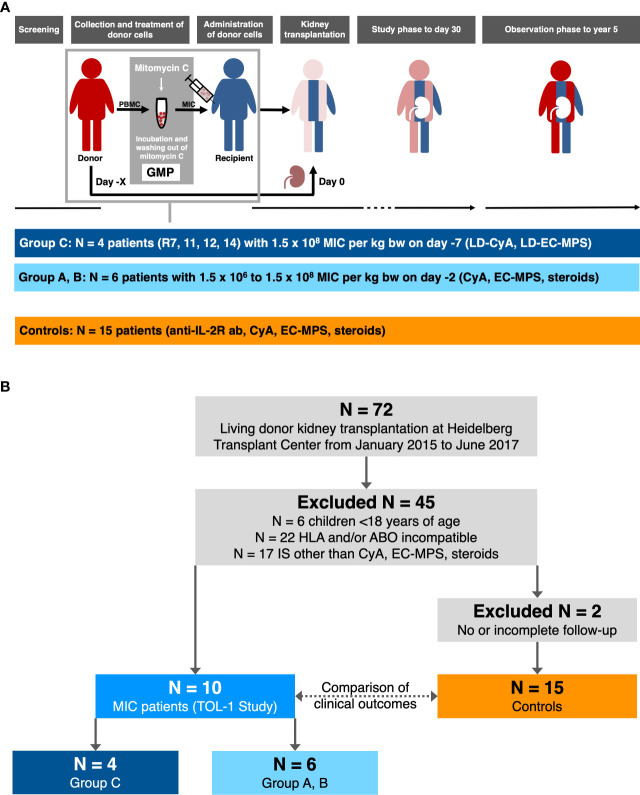
Follow-up of the TOL-1 study and selection of transplanted controls (modified from reference 14 and 15). **(A)** In the TOL-1 study, peripheral blood mononuclear cells (PBMC) were collected from donors and processed under good manufacturing practice (GMP) on day –2 or –7 before kidney transplantation. The final product (MIC) was administered to patients on the same day, approximately 12 hours after donor leukapheresis. Patients received either 1.5 × 10^6^ MIC per kg body weight on day –2 (N = 3, group A) or 1.5 × 10^8^ MIC per kg body weight on day –2 (N = 3, group B) or day –7 (N = 4, group C) before living donor kidney transplantation. After transplantation, patients received cyclosporine A (CyA), enteric-coated mycophenolate sodium (EC-MPS), and corticosteroids (groups A and B, grey bar) with reduced CyA (LD-CyA) and reduced EC-MPS (LD-EC-MPS) and no corticosteroids during follow up in group C patients (blue bar). No anti-IL-2 receptor antibody (anti-IL-2R ab) induction therapy was carried out in MIC patients. The primary outcome measure was the frequency of AE on day 30 with follow-up to year 5 for all patients. **(B)** Controls were selected from a total of 72 patients including the 10 patients who had received pretransplant MIC infusions and who received a living donor kidney graft at Heidelberg Transplant Center from January 01, 2015 to June 30, 2017. After excluding 6 children <18 years of age, 22 patients with an increased immunological risk, and 17 patients on another immunosuppressive therapy than cyclosporine A (CyA), enteric-coated mycophenolate sodium (EC-MPS) and corticosteroids, 10 MIC patients and 17 transplanted controls were identified. Two further transplanted control patients were excluded from the study due to incomplete follow-up so that eventually 15 transplanted controls remained for the current analysis.

From Aug 2015 to Feb 2017, 14 patients and their respective donors were screened for inclusion in the TOL-1 study. Ten patients eventually received MIC intravenously on the day of donor leukapheresis as a single administration as described previously (group A, patient R1-R3: low dose MIC on day -2; group B, patient R4-R6: high dose MIC on day -2; group C, patient R7, R11, R12, R14: high dose MIC on day -7 before surgery; **Figure 1A**) (14). In addition to MIC infusions, patients received immunosuppressive maintenance therapy with cyclosporine A (CyA), enteric-coated mycophenolate sodium (EC-MPS), and corticosteroids without anti-IL-2 receptor antibody induction therapy. MIC patients of group C, who had received the highest cell dose 7 days prior to surgery, showed the strongest donor-specific unresponsiveness (14, 15). In these patients, immunosuppressive maintenance therapy was reduced to lower CyA and lower EC-MPS doses without corticosteroids during follow up beyond day 30.

The control group consisted of adult living donor kidney transplant recipients at the same immunologic risk, i.e., ABO-compatible and non-sensitized, who received the same immunosuppressive maintenance therapy, i.e., CyA, EC-MPS, and corticosteroids, and who had sufficient follow-up. Overall, 15 patients transplanted during the same period met these criteria (**Figure 1B**). These patients were not selected for inclusion into the TOL-1 study since they were transplanted shortly before study start (N=3), or shortly after study end (N=2), since they could not speak German language (N=1), or due to lack of consent (N=4). Three patients had a contraindication for study inclusion, i.e., preexisting immunosuppressive therapy (N=1) or prior cured malignancy (N=2). Two patients were screened for study inclusion but excluded for safety reasons because they were found to have (non-HLA) antibodies (e.g. anti-erythrocyte antibodies) that could harm the patient during MIC infusions.

Baseline patient characteristics are summarized in **Table 1**, and CyA trough levels as well as EC-MPS and corticosteroid doses are given in **Supplemental Figure 1**, for MIC patients and controls separately.

**Table 1:** Baseline patient characteristics (modified from references 14 and 15).

	Controls (N = 15)	MIC			*P* ^A^
		Group A, B (N = 6)	Group C (N = 4)	Total cohort (N = 10)	
Patient					
Age (years) – median (range)	51 (28-67)	34 (22-59)	46 (29-50)	40 (22-59)	0.053
Male sex – N (%)	12 (80)	5 (83)	3 (75)	8 (80)	1.0
Cause of ESRD – N (%)					0.86
Vascular Diabetes mellitus Glomerulonephritis Polycystic kidney disease Other or unknown	0 (0) 0 (0) 6 (40) 3 (20) 6 (40)	0 (0) 0 (0) 3 (50) 1 (17) 2 (33)	0 (0) 0 (0) 2 (50) 1 (25) 1 (25)	0 (0) 0 (0) 5 (50) 2 (20) 3 (30)	
Living donor					
Living related – N (%)	7 (47)	4 (67)	4 (100)	8 (80)	0.21
Age (years) – median (range)	53 (25-73)	52 (46-61)	55 (42-57)	54 (42-61)	0.99
Male sex – N (%)	6 (40)	1 (17)	2 (50)	3 (30)	0.69
Serological data					
CMV serologic status – N (%)					0.84
Donor negative, recipient negative Donor negative, recipient positive Donor positive, recipient positive Donor positive, recipient negative	5 (33) 1 (7) 6 (40) 3 (20)	2 (33) 0 (0) 3 (50) 1 (17)	1 (25) 0 (0) 2 (50) 1 (25)	3 (30) 0 (0) 5 (50) 2 (20)	
EBV virus serologic status – N (%)					0.40
Donor negative, recipient negative Donor negative, recipient positive Donor positive, recipient positive Donor positive, recipient negative	0 (0) 1 (7) 14 (93) 0 (0)	0 (0) 0 (0) 6 (100) 0 (0)	0 (0) 0 (0) 4 (100) 0 (0)	0 (0) 0 (0) 10 (100) 0 (0)	
Immunological data					
HLA-A, -B, -DR mismatches – N (%)					0.54
0 1 2 3 4 5 6	2 (13) 0 (0) 3 (20) 4 (27) 0 (0) 6 (40) 0 (0)	1 (17) 0 (0) 0 (0) 3 (50) 0 (0) 2 (33) 0 (0)	1 (25) 1 (25) 1 (25) 1 (25) 0 (0) 0 (0) 0 (0)	2 (20) 1 (10) 1 (10) 4 (40) 0 (0) 2 (20) 0 (0)	
Patients with sensitizing events – N (%) Transplantation Blood transfusion Pregnancy	3 (20) 0 (0) 2 (13) 1 (7)	0 (0) 0 (0) 0 (0) 0 (0)	2 (50) 0 (0) 2 (50) 0 (0)	2 (20) 0 (0) 2 (20) 0 (0)	1.0
PRA (%) – median (range)					–
CDC T cell (- DTT) CDC T cell (+ DTT) CDC B cell (- DTT) CDC B cell (+ DTT) Single Antigen Bead (HLA class I) Single Antigen Bead (HLA class II)	0 (0-0) 0 (0-4) 0 (0-15) 4 (0-37) 0 (0-0) 0 (0-9)	0 (0-1) 0 (0-0) 0 (0-12) 0 (0-35) 0 (0-2) 0 (0-2)	2 (0-6) 2 (0-4) 0 (0-4) 2 (0-4) 0 (0-2) 3 (0-9)	0 (0-6) 0 (0-4) 0 (0-12) 2 (0-35) 0 (0-2) 1 (0-9)	
Patients with pre-transplant DSA – N (%)	0 (0)	0 (0)	0 (0)	0 (0)	1.0
Baseline immunosuppressive therapy					
Anti-IL-2 receptor antibody induction Cyclosporine A, enteric-coated mycophenolate sodium, corticosteroids	15 (100) 15 (100)	0 (0) 6 (100)	0 (0) 4 (100)	0 (0) 10 (100)	<0.001 1.0

^A^For the comparison of MIC patients, Total cohort (N = 10) versus Controls (N = 15). CDC, complement-dependent cytotoxicity; CMV, cytomegalovirus; DSA, donor-specific HLA-A, -B, -DR, -DQ antibodies at a cutoff of 1,000 MFI; DTT, dithiothreitol; EBV, Epstein-Barr virus; ESRD, end-stage renal disease; HLA, human leukocyte antigen; PRA, panel-reactive antibody.

### Modified immune cell product manufacturing

The MIC product was based on donor peripheral blood mononuclear cells obtained by unstimulated leukapheresis that were processed under good manufacturing practice conditions as previously described (14, 15). In brief, donor blood cells were incubated with mitomycin C. After washing out mitomycin C and the appropriate quality controls, the product was released for infusion to patients.

### Further investigations

Human leukocyte antigen antibody detection (Luminex, ELISA and CDC) and crossmatch techniques (ELISA and CDC), lymphocyte proliferation assay (mixed lymphocyte culture), determination of lymphocyte subsets in peripheral blood (flow cytometry), and determination of cytokine and chemokine levels in plasma (Luminex Performance Assays, R&D Systems, Wiesbaden, Germany) were performed as previously described (14, 15).

### Statistics

In the text and tables, continuous data are summarized as the median and range and categorical data as absolute and relative frequencies. In figures, the results are presented as individual measurements (and the median) or the median and interquartile range. For comparison of MIC patients (total cohort) with the control group, the two group Mann Whitney U test and the Chi-Square test were used as deemed appropriate. Statistical comparisons of different MIC groups were not performed due to low patient numbers. Graft survival, *de novo* DSA-free survival and opportunistic infection-free survival were calculated according to the Kaplan-Meier method.

### Study approval

The TOL-1 study was reviewed and approved by the ethics committee of the University of Heidelberg, Heidelberg, Germany, and the Paul-Ehrlich Institute, Langen, Germany (ethics number: AFmo-549/2014; Paul-Ehrlich Institute, Vorlagen-Nr. 2252/01; EudraCT number: 2014-002086-30; Clinicaltrials.gov identifier: NCT02560220). The study was performed in compliance with the provisions of the Declaration of Helsinki and Good Clinical Practice guidelines. Written informed consent was obtained from participants before inclusion in the study. During follow-up, patient data and biomaterials were collected and analyzed retrospectively according to protocols approved by the ethics committee of the University of Heidelberg (ethics numbers: 082/2005, 083/2005, S-395/2011, and S-225/2014).

## Results

### Comparison of clinical outcomes of MIC patients to outcomes of matched controls

This is the 5-year follow up of 10 patients who participated in a phase I clinical trial of donor-derived MIC therapy for individualized immunosuppression in living donor kidney transplant recipients (TOL-1 Study, **Figure 1A**) (14, 15). MIC patients were retrospectively compared to 15 matched control patients on standard immunosuppressive therapy but without MIC infusions (**Figure 1B**). MIC patients showed similar baseline characteristics but differed from controls who had received anti-IL-2 receptor antibody induction therapy with basiliximab at days 0 and 4 after transplantation instead of MIC infusions (**Figure 1A** and **Table 1**). In addition, MIC patients tended to be younger (**Table 1**) and MIC patients of group C had lower immunosuppressive maintenance therapy than transplanted controls beyond day 30 after transplantation (**Supplemental Figure 1**).

**Figure 2:**
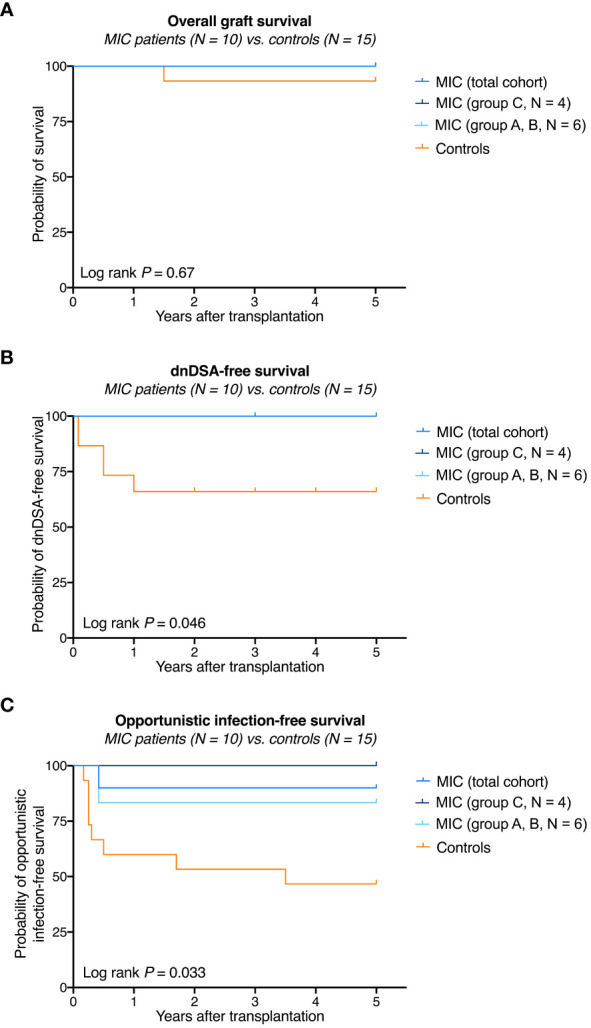
Survival rates in MIC patients compared to transplanted controls. **(A)** Overall graft survival in MIC patients was not significantly different from survival in transplanted controls. One transplanted control patient died on post-operative day 531 from severe infectious complications including mucormycosis caused by *Rhizopus microsporus*. **(B)** None of the 10 MIC patients but 5 of 15 transplanted control patients developed *de novo* donor-specific human leukocyte antigen antibodies (dnDSA) against HLA-A, -B, -DR, or -DQB antigens at a cutoff of 1,000 MFI. **(C)** Opportunistic infection-free survival (i.e., no CMV replication >1.000 IU/mL, no BKV replication >10.000 copies/mL, no other opportunistic infections) was significantly higher in MIC patients compared to transplanted controls. The log rank *P* value is given for the comparison of MIC (total cohort) versus transplanted controls.

**Figure 3:**
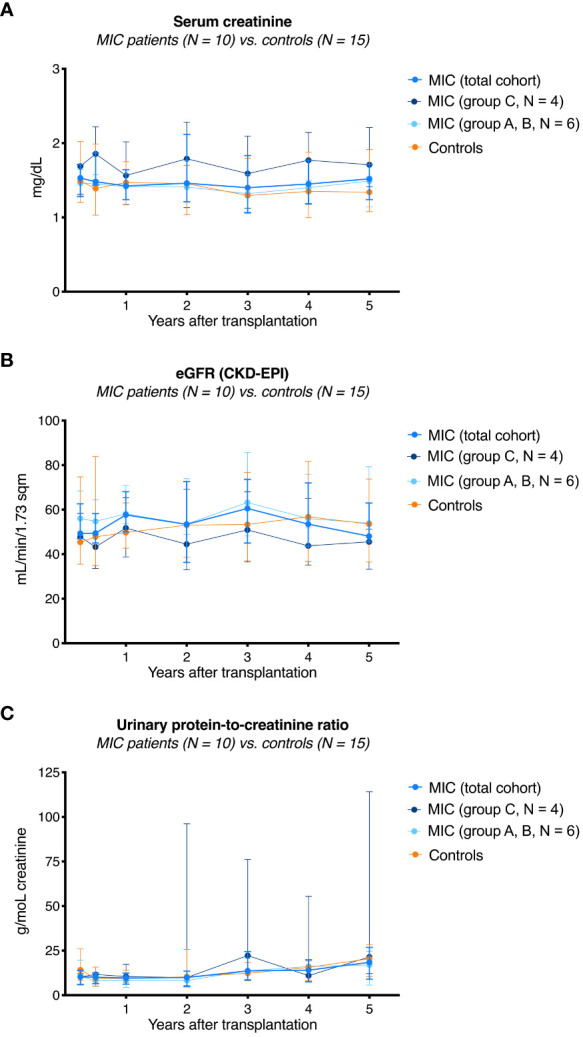
Kidney function and integrity in MIC patients compared to transplanted controls. Serum creatinine **(A)**, eGFR according to the CKD-EPI formular **(B)**, and urinary protein excretion **(C)** in 10 MIC patients were not different to the numbers in 15 transplanted controls.

Overall graft survival in MIC patients was not significantly different from survival in transplanted controls (log rank *P* = 0.67, **Figure 2A**). One transplanted control patient died on post-operative day 531 from severe infectious complications including mucormycosis caused by *Rhizopus microsporus.* Five-year kidney graft function and urinary protein excretion in MIC patients showed a median serum creatinine of 1.52 mg/dL (range 1.12–2.37 mg/dL), a median estimated glomerular filtration rate (eGFR) according to CKD-EPI of 48 mL/min/1.73 m^2^ (range 30–87 mL/min/1.73 m^2^), and a median urinary protein excretion of 18 g/moL creatinine (range 3–144 g/moL creatinine), all not significantly different from transplanted controls (median 1.34 mg/dL, range 0.78–3.61 mg/dL, *P*=0.61, median 54 mL/min/1.73 m^2^, range 18–115 mL/min/1.73 m^2^, *P*=0.70, and median 20 g/moL creatinine, range 10–30 g/moL creatinine, *P*=0.83, respectively, **Figure 3**).

During follow up to year 5, no acute rejection episodes were noted in MIC patients and transplanted controls (**Table 2**). MIC patients showed no evidence of *de novo* DSA though they had received up to 1.34 x10^10^ nucleated donor blood cells 2 or 7 days before surgery and, in case of group C patients, were on low immunosuppressive maintenance therapy thereafter. In comparison, a total of 5 of 15 (33%) transplanted controls on full immunosuppressive therapy developed *de novo* DSA during the 5-year follow up (log rank *P* = 0.046, **Figure 2B** and **Table 2**).

**Table 2:** Outcomes and complications in MIC patients compared to transplanted controls out to year 5.

	Controls (N = 15)	MIC			*P* ^A^
		Group A, B (N = 6)	Group C (N = 4)	Total cohort (N = 10)	
Pat. with BCR – N (%) Rejection episodes – N Acute TCMR (≥BANFF IA) Chronic active TCMR Acute ABMR Chronic active ABMR	1 (7) 1 0 0 0 1	1 (17) 1 0 1 0 0	0 (0) 0 0 0 0 0	1 (10) 1 0 1 0 0	1.0
Pat. with *de novo* DSA – N (%)	5 (33)	0 (0)	0 (0)	0 (0)	0.061
Pat. with DGF – N (%)	0 (0)	0 (0)	0 (0)	0 (0)	1.0
Pat. with opportunistic infections – N (%) Infectious episodes – N Pneumonia CMV reactivation > 1,000 IU/mL BKV replication > 10,000 copies/mL BKV-associated nephropathy Other	8 (53) 13 4 4 2 0 3	1 (17)^B^ 1^B^ 0 1^B^ 0 0 0	0 (0) 0 0 0 0 0 0	1 (10)^B^ 1^B^ 0 1^B^ 0 0 0	0.041
Pat. with non-opportunistic infections – N (%) Infectious episodes – N CVC-associated infection Urinary tract infection Post-operative wound infection Pneumonia Influenza A/B, COVID-19 Other or unknown	13 (87) 43 1 16 1 7 3 15	4 (67) 9 1 6 0 0 1 1	3 (75) 15 0 8 1 1 1 4	7 (70) 24 1 14 1 1 2 5	0.36
Pat. with multi-resistant bacteria – N (%)	3 (20)	0 (0)	0 (0)	0 (0)	0.25
Pat. with rehospitalization – N (%) Total rehospitalizations – N	12 (80) 38	6 (100) 10	4 (100) 16	10 (100) 26	0.25
Pat. with PTLD or malignancy – N (%)	1 (7)	0 (0)	0 (0)	0 (0)	1.0
Pat. with cardiovascular events – N (%)	1 (7)	0 (0)	0 (0)	0 (0)	1.0
Pat. with surgical complications^C^ – N (%) Bleeding Wound healing disturbances Urinary leakage or stenosis Lymphocele Hernia Other	6 (40) 0 0 2 2 1 1	2 (33) 0 2 0 0 0 0	2 (50) 1 0 1 0 0 0	4 (40) 1 2 1 0 0 0	1.0
Total antihypertensive therapeutic intensity score (TIS) – median (range)					
Year 1 Year 3 Year 5	0.9 (0-2.8) 1.0 (0-2.3) 0.9 (0-2.5)	1.1 (0.3-1.5) 1.1 (0.3-1.8) 1.3 (0.3-1.8)	1.0 (0.2-3.0) 1.0 (0.4-2.5) 1.5 (0.4-2.8)	1.0 (0.2-3.0) 1.0 (0.3-2.5) 1.3 (0.3-2.8)	0.94 0.87 0.65
NODAT^C^ – N (%)	2 (13)	0 (0)	0 (0)	0 (0)	0.50

^A^For the comparison of MIC patients, Total cohort (N = 10) versus Controls (N = 15). ^B^This episode was identified retrospectively during review of a letter from the primary physician. CMV reactivation had resolved without treatment. ^C^Requiring treatment. ABMR, antibody-mediated rejection; BCR, biopsy-confirmed rejection; BKV, BK virus; CMV, cytomegalovirus; CVC, central venous catheter; DGF, delayed graft function; DSA, donor-specific HLA-A, -B, -DR, -DQ antibodies at a cutoff of 1,000 MFI; NODAT, new-onset diabetes after transplantation; PTLD, post-transplant lymphoproliferative disease; TCMR, T-cell-mediated rejection.

One MIC patient was retrospectively found to have mild CMV reactivation 5 months after surgery, which resolved without further treatment (patient R1 of group A). Otherwise, no opportunistic infections were detected in the MIC patients during rigorous screening. In comparison, 8 transplanted controls (53%) developed opportunistic infections during follow up including 4 episodes of opportunistic pneumonia and 6 episodes of CMV- or BKV-replication (log rank *P* = 0.033, **Figure 2C** and **Table 2**). During follow-up to year 5, 2 of 15 controls (13%) and 1 of 10 MIC patients (10%) suffered from mild courses of COVID-19 infection (*P* = 1.0, data not shown). Delayed graft function (*P*=1.0), rehospitalization (*P*=0.25), PTLD or malignancy (*P*=1.0), cardiovascular events (*P*=1.0), surgical complications (*P*=0.70), total antihypertensive therapeutic intensity score at year 5 (*P*=0.65), and new-onset diabetes after transplantation (*P*=0.52) did not differ significantly between MIC patients and transplanted controls (**Table 2**).

When only 10 of the 15 patients who had no formal contraindication to enrollment in the TOL-1 Study (N = 3) or were excluded during screening (N = 2) were analyzed, virtually the same results were obtained (**Supplemental Figure 2**). However, due to the smaller number of patients, some comparisons failed to reach statistical significance.

### Immunological findings in MIC patients

Based on preclinical data and the one- and three-year results of this phase I study (14, 15), it was clear that group C patients, who had received the highest MIC number seven days before surgery, showed the strongest donor-specific immunosuppression (**Supplemental Figure 3**). Therefore, these patients underwent comprehensive immunologic monitoring during follow-up. However, 5-year clinical data suggested that patients in group A, B might also have a graft-protective phenotype, so additional immunologic data were collected on these patients.

**Figure 4:**
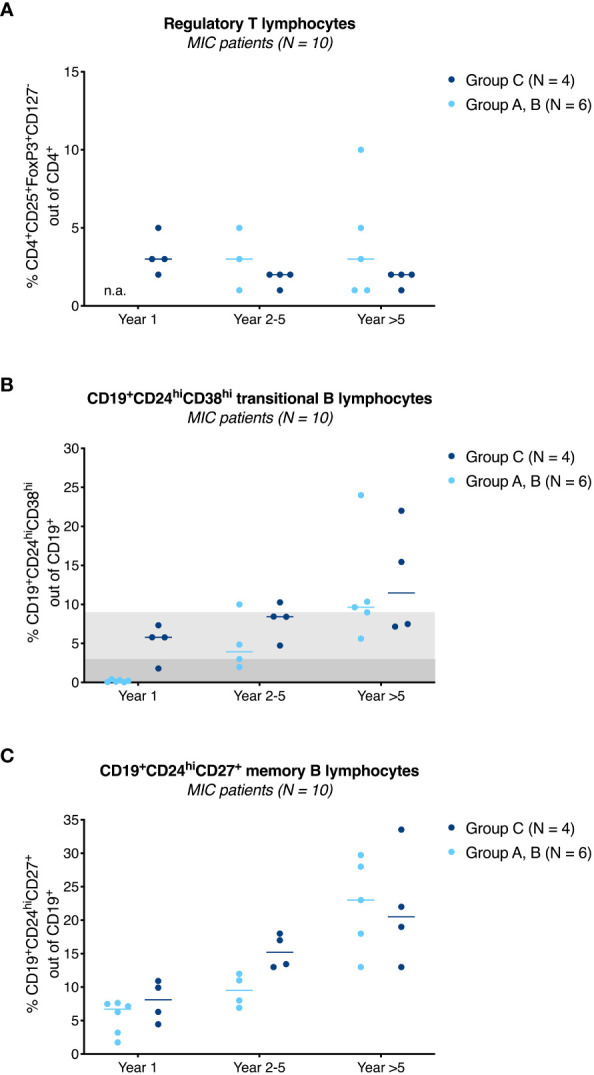
Immunological results of MIC patients. Percentage of CD4^+^CD25^+^FoxP3^+^CD127^-^ regulatory T lymphocytes **(A)**, CD19^+^CD24^hi^CD38^hi^ transitional B lymphocytes **(B)**, and CD19^+^CD24^hi^CD27^+^ memory B lymphocytes **(C)** in MIC patients of group A, B (●) and group C (●) at year 1, year 2-5 and beyond year 5 after surgery are shown. Individual measurements and the median are given. In contrast to regulatory T lymphocytes **(A)**, CD19^+^CD24^hi^CD38^hi^ transitional B lymphocytes in MIC patients of group C remained above baseline values during follow-up **(B)**. MIC patients of group A, B had lower percentages of this cell type during the first year after transplantation (14), but percentages increased over time and appeared to approach the percentages of MIC patients of group C during follow-up. Values usually found in transplanted patients on triple drug immunosuppressive therapy are highlighted in dark grey (18, 19), values usually found in operational tolerant patients in light grey (16, 17) **(B)**. From year 1 onwards, there was a steady increase of CD19^+^CD24^hi^CD27^+^ memory B lymphocytes with no obvious differences between MIC patients of group A-C **(C)**. In group A, B, only a limited number of samples was available for analysis. Regulatory T lymphocytes, year 1: not available (n.a.), year 2-5: N = 3, year >5: N = 5 **(A)**. Regulatory B lymphocytes, year 2-5: N = 4, year >5: N = 5 **(B, C)**. Analysis of transitional and memory B lymphocytes at year 1 was performed from frozen cells all other flow cytometric analyses were performed from fresh cells.

**Figure 4A** shows the evolution of CD4^+^CD25^+^FoxP3^+^CD127^-^ regulatory T lymphocytes as percentage of the total CD4^+^ lymphocyte pool for MIC patients stratified by group to year 5 after surgery. Regulatory T lymphocyte percentages were stable at a median of 2-3% (range 1–10%) during follow-up with no obvious differences between groups or compared to baseline (median 3%, range 2-4%, data not shown). In contrast to regulatory T lymphocytes, analysis of CD19^+^CD24^hi^CD38^hi^ transitional B lymphocytes showed persistently high frequencies of this regulatory cell type in group C patients beyond year 5 with a median of 7% (range 7–22%, **Figure 4B**). These percentages were in the range reported for operationally tolerant [3–8%, indicated in **Figure 4B** in light grey (16, 17)], but higher than reported for stable immunosuppressed kidney transplant recipients [0–5%, indicated in **Figure 4B** in dark grey (18, 19)]. Unlike in group C patients, the percentages of CD19^+^CD24^hi^CD38^hi^ transitional B lymphocytes in MIC patients of group A, B were low in the first year after surgery but then increased over time and appeared to approach the percentages of MIC patients of group C after the 5^th^ year (**Figure 4B**) (14). Interestingly, numbers of (regulatory) CD19^+^CD24^hi^CD38^hi^ transitional B lymphocytes seemed to closely follow the numbers of (alloreactive) activated CD8^+^DR^+^ T lymphocytes (**Supplemental Figure 4**). For example, patient R11, in whom immunosuppressive therapy was reduced to low CyA monotherapy with a trough level of only 64 μg/L during an infectious episode showed an increase in CD8^+^DR^+^ T lymphocytes from day 720 that was mimicked by the course of CD19^+^CD24^hi^CD38^hi^ transitional B lymphocytes (**Supplemental Figure 4B**) and serum IL-10 levels (data not shown). Enteric-coated mycophenolate sodium was reinstituted at 720 mg/day thereafter and the patient had an uneventful subsequent course.

A second regulatory B lymphocyte subset, CD19^+^CD24^hi^CD27^+^ memory B lymphocytes, increased over time after transplantation with no obvious differences between MIC patients of group A-C (**Figure 4C**).

Except for HLA antibody monitoring (see above), no immunological analyses were available for transplanted controls.

## Discussion

We have recently shown that a single pretransplant infusion of MIC led to long-lasting donor-specific unresponsiveness (14, 15, 20, 21). This was accompanied by an increased frequency of regulatory B lymphocytes and a consensus gene expression signature of operational tolerance. The question arises whether this intervention, together with reduced immunosuppression, was sufficient to effectively prevent the occurrence of an alloimmune response while reducing the deleterious side effects of conventional drug therapy. To test this hypothesis, the clinical outcomes of the 10 MIC patients were compared to the outcomes of 15 standard-risk recipients who received regular triple-drug maintenance therapy. Neither acute rejections nor DSA were detected in MIC patients, although they had received mononuclear donor blood cells 2 or 7 days before surgery. We also found fewer opportunistic infections in MIC patients when compared to transplanted controls, most likely as a result of the reduced immunosuppressive therapy. These results held strong when comparing MIC patients only to ten transplanted controls who had no formal contraindication for inclusion into the TOL-1 study. Most strikingly, MIC patients continued to show absent anti-donor T cell reactivity and high transitional B lymphocyte numbers out to year 5 which might be considered a proof of continued donor-specific unresponsiveness.

In our current analysis, none of the 10 MIC patients showed evidence of DSA against HLA-A, -B, -DR, or -DQB antigen mismatches during the 5-year follow-up period. The absence of DSA after but also on day -1 before transplantation is even more remarkable considering that MIC patients had received up to 1.34 x10^10^ nucleated donor blood cells 2 or 7 days before surgery. Due to the introduction of the donor antigen already 7 days before transplantation, one would normally expect a sensitizing effect. A graft-protective effect similar to the one observed in our study was also observed by Marti et al. when infusing unmodified mononuclear cells twice before living donor kidney transplantation, but at the expense of sensitizing 6 of 61 (10%) patients by administering cells bearing donor HLA on their surface before surgery (22). In our study, no such sensitization was detected before transplantation, which is most likely due to the modification process of the cells, but also to the infusion timing at day -7 before transplantation, which did not allow sufficient time for allosensitization. In comparison, a higher proportion of 5 of 15 (33%) transplanted controls developed DSA during 5-year follow-up, which is considerably higher than in MIC patients but also slightly higher than reported in the literature for transplanted patients at comparable immunologic risk (23, 24). In a study by Wiebe et al, only 15% of patients overall developed DSA at a median of 6.2 years post-transplant (25). These DSA, especially when capable of activating complement, bind to the endothelium of the allograft and lead to antibody-mediated allograft injury (25, 26). Explanations for the observed differences between transplanted controls in our study and findings from the literature could be the use of cyclosporine A (instead of tacrolimus) or the stringent routine screening in our patients, in which DSA were detectable only in a low range of up to 2,052 MFI in 3 of 5 patients.

Infections, malignancies, and cardiovascular events are the major causes of premature death in immunocompromised kidney transplant recipients (27–30). In the Swiss Transplant Cohort Study, a total of 1964 clinically relevant infections, mostly bacterial and viral, were identified in 1612 kidney transplant recipients during the first year after surgery (28). Overall, 81% of patients had an infectious event, and 53% of patients had at least one clinically relevant infection. A total of 12 patients eventually died from infection. The One study recently demonstrated that cell therapy approaches combined with reduced immunosuppression are capable of effectively reducing infectious episodes (10). The incidence of serious adverse events related to infection and infestation was nearly six times higher in the control trial than in the combined cell therapy trials, and this increase was primarily due to a higher number of viral infections in the control trial (10). Likewise, we found in our study an increased frequency of opportunistic infections, mostly viral, and a trend towards more pneumonia episodes from any cause (opportunistic and non-opportunistic combined) in matched control patients when compared to MIC patients on reduced immunosuppressive therapy. We cannot judge on the effect of our approach on malignancy and cardiovascular events since only 1 episode of each was detected during the study, notably both in transplanted control patients on full immunosuppressive therapy.

We admit that our study has limitations. We show 5-year follow-up of only 10 patients from a 30-day single-arm phase I clinical trial, and only 4 patients had received the highest MIC number 7 days before surgery, which was considered most effective. The 15 transplanted control patients were thoroughly matched but were selected retrospectively, and a potential selection bias toward better outcomes in MIC patients cannot be excluded. In addition, the control patients had basiliximab induction therapy and, because of the nature of the study, stronger immunosuppressive maintenance therapy, at least compared with MIC patients of group C. Another limitation arises from the fact that the anti-donor T-cell response and flow cytometry analyses were available only in MIC patients but not in transplanted controls. Nevertheless, the concomitant lower infection rates and a lower incidence of DSA in MIC patients suggest a potential clinical relevance of this cell therapy product when administered together with reduced conventional immunosuppressive therapy.

In conclusion, MIC infusions prior to living donor kidney transplantation were shown in this small series to result in donor-specific unresponsiveness that effectively controls alloimmune responses. One would expect that this treatment would offer an opportunity to reduce the side effects of conventional chemical immunosuppression. These results are very promising and suggest that MIC therapy is a potential new effective treatment option for kidney transplant recipients. A Phase IIb study is currently underway to test the hypothesis that a larger series of patients treated with MIC and a reduced immunosuppressive drug regimen will show the same positive effects (EudraCT No. 2021-000561-33, ClinicalTrials.gov Identifier: NCT05365672) (31).

## Data availability statement

The original contributions presented in the study are included in the article/**Supplementary Material**. Further inquiries can be directed to the corresponding author.

## Ethics statement

The studies involving human participants were reviewed and approved by Ethics committee of the University of Heidelberg, Heidelberg, Germany. The patients/participants provided their written informed consent to participate in this study.

## Author contributions

MSa, CMo, CK, GO, CS, DC, MZ, MSm, PT, and AS designed the study. MSa, CMo, and MZ recruited participants. CK, LW, AH-K, MSm, and AS produced MIC under GMP conditions and performed quality controls of the products. MSa, CMo, FK, CSp, LB, CN, CMa, LS, and MSa collected the data. MSa, CMo, LW, CK, TT, SS, LP, NE, CSü, GP, AM, CSc, RW, PS, UM, VS, MK, SK, MF, MSt, IH, A-IK, BK, GB, CM-T, JR, MSm, PT, AS, and VD analyzed the data. MSa, CMo, GO, TT, CSü, MZ, MSm, PT, AS, and VD interpreted the data. MSa and CMo designed the figures. MSa, CMo, GO, and VD wrote the first draft of the manuscript, and all authors revised it critically and approved the final version. All authors contributed to the article and approved the submitted version.
