# Supplementary material for: Five-year follow-up of a phase I trial of donor-derived modified immune cell infusion in kidney transplantation
Source: Front Immunol. 2023 Jul 11;14:1089664. doi: 10.3389/fimmu.2023.1089664 (PMC10361653; doi:10.3389/fimmu.2023.1089664)
Supplement: Supplementary file 1 [file DataSheet_1.pdf]

Supplementary Material

Five-year follow-up of a phase I trial of donor-derived modified immune cell infusion in kidney transplantation

Matthias Schaier, Christian Morath, Lei Wang, Christian Kleist, Gerhard Opelz, Thuong Hien Tran, Sabine Scherer, Lien Pham, Naruemol Ekpoom, Caner Süsal, Gerald Ponath, Florian Kälble, Claudius Speer, Louise Benning, Christian Nusshag, Christoph F. Mahler, Luiza Pego da Silva, Claudia Sommerer, Angela Hückelhoven-Krauss, David Czock, Arianeb Mehrabi, Constantin Schwab, Rüdiger Waldherr, Paul Schnitzler, Uta Merle, Vedat Schwenger, Markus Krautter, Stephan Kemmner, Michael Fischereder, Manfred Stangl, Ingeborg A. Hauser, Anna-Isabelle Kälsch, Bernhard K. Krämer, Georg A. Böhmig, Carsten Müller-Tidow, Jochen Reiser, Martin Zeier, Michael Schmitt, Peter Terness, Anita Schmitt, Volker Daniel

M Schaier and C Morath are co-first authors, P Terness, A Schmitt and V Daniel are co-senior authors.

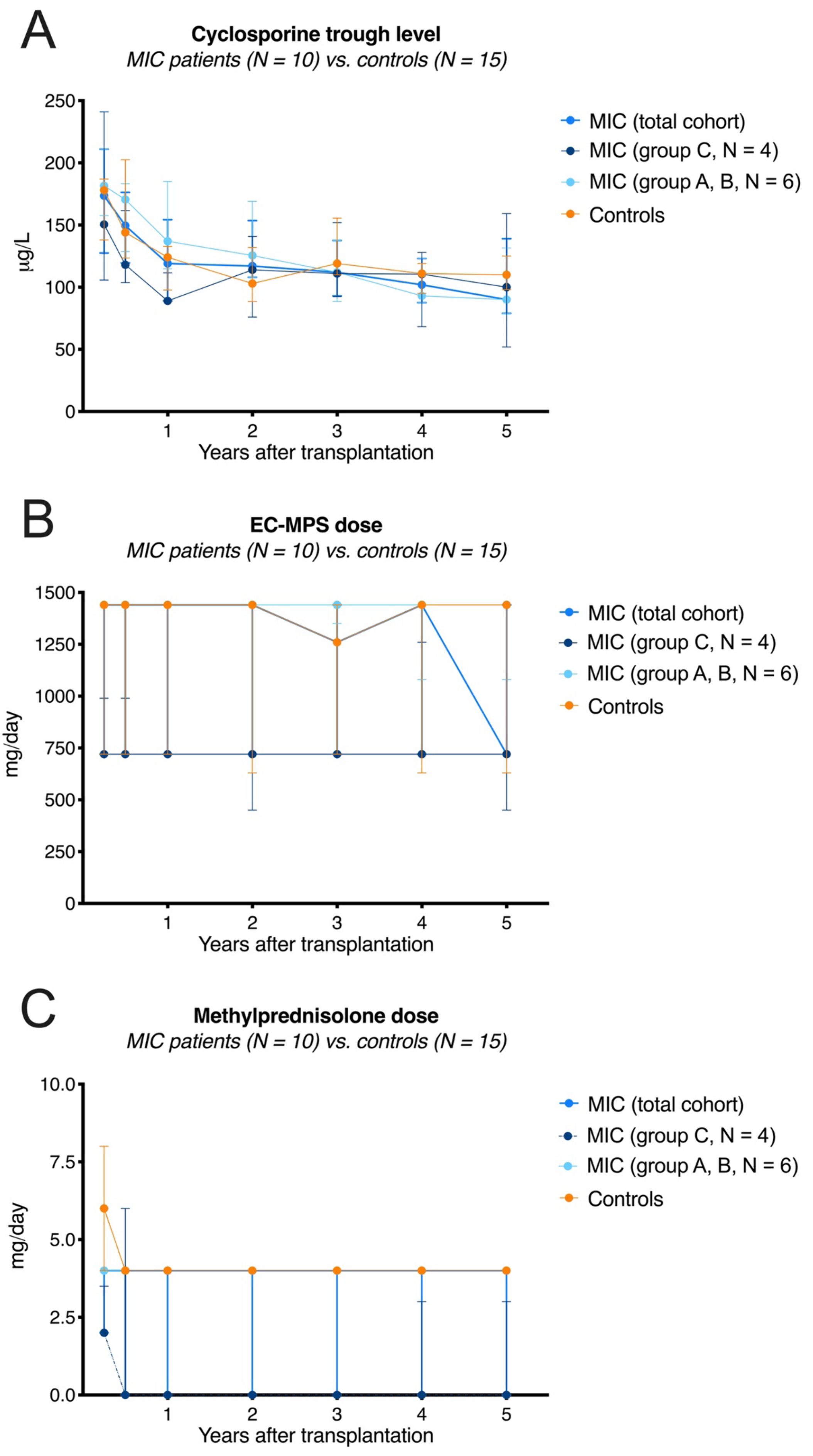
A
Cyclosporine trough level
MIC patients (N = 10) vs. controls (N = 15)
µg/L
250
200
150
100
50
0
1
2
3
4
5
Years after transplantation
MIC (total cohort)
MIC (group C, N = 4)
MIC (group A, B, N = 6)
Controls
B
EC-MPS dose
MIC patients (N = 10) vs. controls (N = 15)
mg/day
1500
1250
1000
750
500
250
0
1
2
3
4
5
Years after transplantation
MIC (total cohort)
MIC (group C, N = 4)
MIC (group A, B, N = 6)
Controls
C
Methylprednisolone dose
MIC patients (N = 10) vs. controls (N = 15)
mg/day
10.0
7.5
5.0
2.5
0.0
1
2
3
4
5
Years after transplantation
MIC (total cohort)
MIC (group C, N = 4)
MIC (group A, B, N = 6)
Controls

Supplementary Figure 1: Immunosuppressive therapy in MIC patients compared to transplanted controls

Cyclosporine A trough level (A), as well as total daily enteric-coated mycophenolate sodium (EC-MPS) (B) and methylprednisolone dose (C) in 10 MIC patients compared to 15 transplanted controls. MIC patients of group C received low doses of cyclosporine A and enteric-coated mycophenolate sodium (EC-MPS), and no corticosteroids during follow-up.

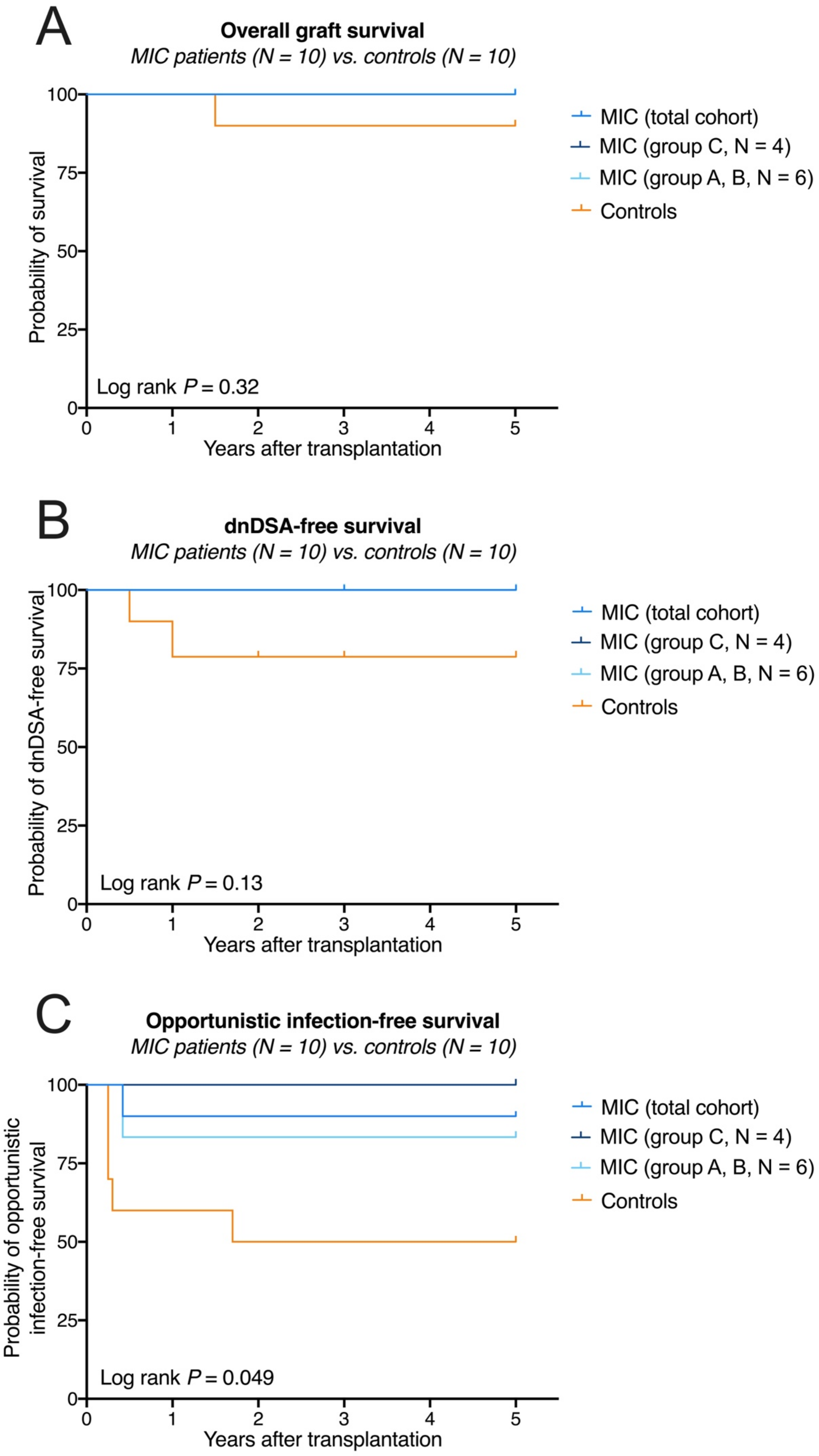
A
Overall graft survival
MIC patients (N = 10) vs. controls (N = 10)
100
75
50
25
0
Probability of survival
Log rank P = 0.32
0 1 2 3 4 5
Years after transplantation
MIC (total cohort)
MIC (group C, N = 4)
MIC (group A, B, N = 6)
Controls
B
dnDSA-free survival
MIC patients (N = 10) vs. controls (N = 10)
100
75
50
25
0
Probability of dnDSA-free survival
Log rank P = 0.13
0 1 2 3 4 5
Years after transplantation
MIC (total cohort)
MIC (group C, N = 4)
MIC (group A, B, N = 6)
Controls
C
Opportunistic infection-free survival
MIC patients (N = 10) vs. controls (N = 10)
100
75
50
25
0
Probability of opportunistic infection-free survival
Log rank P = 0.049
0 1 2 3 4 5
Years after transplantation
MIC (total cohort)
MIC (group C, N = 4)
MIC (group A, B, N = 6)
Controls

Supplementary Figure 2: Survival rates in MIC patients compared to 10 transplanted controls

When comparing MIC patients to 10 transplanted controls who had no formal contraindication to enrollment in the TOL-1 study (N = 3) or were excluded during screening (N = 2) the results were comparable to results obtained in the greater cohort of 15 patients.

The log rank *P* value is given for the comparison of MIC (total cohort) versus transplanted controls.

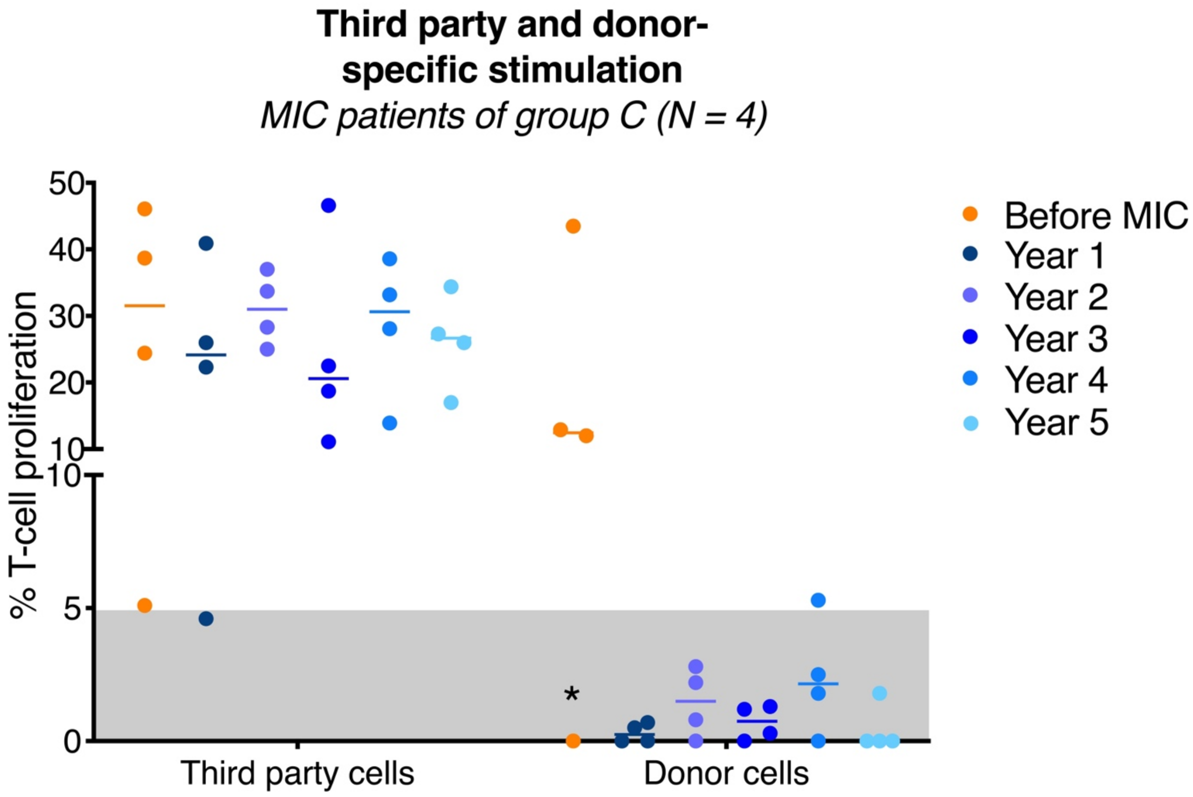

Supplementary Figure 3: Third party and donor-specific lymphocyte response of MIC-treated patients of group C in vitro

(A) Third party and donor-specific stimulation of peripheral blood lymphocytes from MIC patients of group C in vitro at year 1 (●), year 2 (●), year 3 (●), year 4 (●), and year 5 (●) compared to before MIC treatment on day -7 (●). Individual measurements and median are shown. Values outside the normal range for healthy individuals are highlighted in grey. The findings indicate preserved immunological responsiveness of recipient T lymphocytes against irradiated third party cells with reduced responsiveness to donor cells after transplantation compared to before transplantation and MIC infusion. Stimulatory cells consisted of irradiated allogeneic peripheral blood mononuclear cells. T lymphocyte proliferation was assessed by carboxyfluorescein succinimidyl ester (CFSE) staining. * = patient R14 had 0 HLA A-, B-, DR-mismatches with the donor.

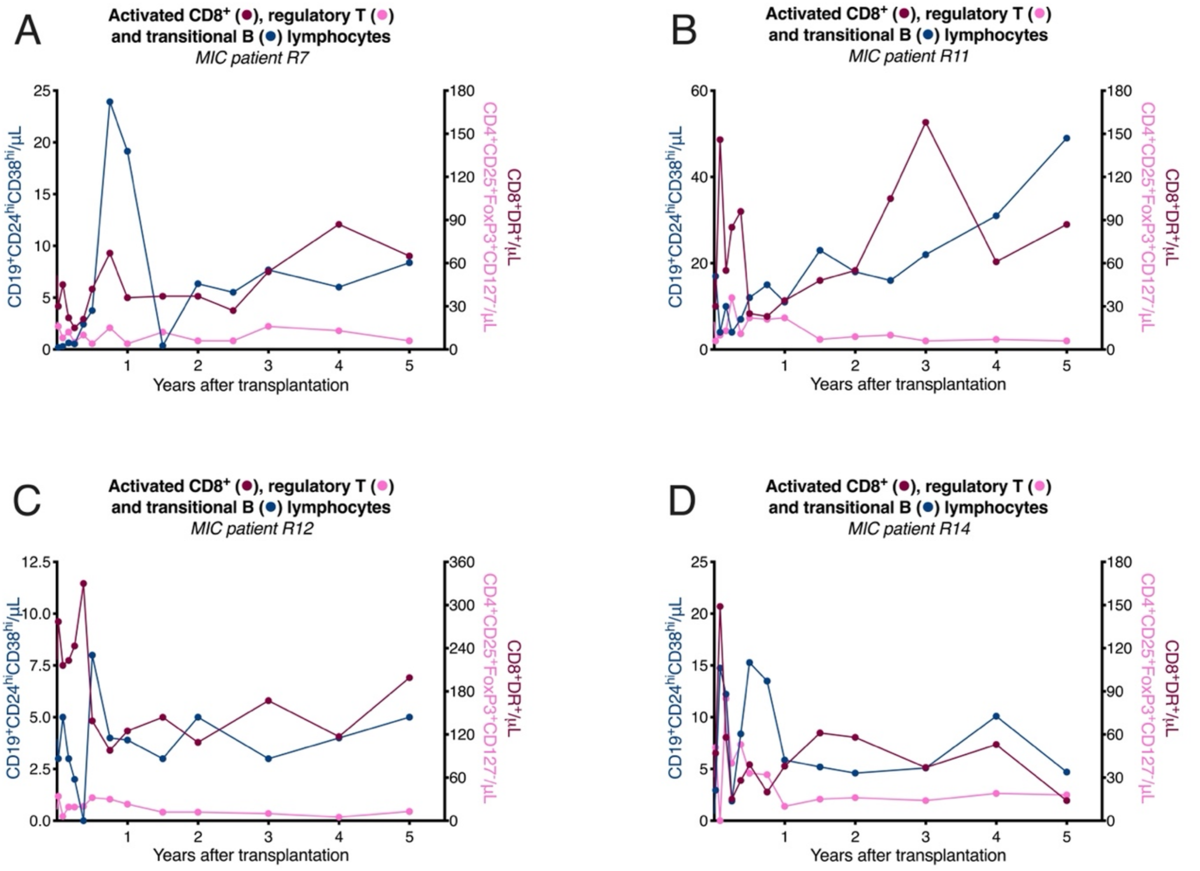

Supplementary Figure 4: Course of activated CD8$^+$, regulatory T and transitional B lymphocytes in individual MIC patients of group C

Individual measurements for CD19$^+$CD24^{hi}CD38^{hi} transitional B lymphocytes, CD4$^+$CD25$^+$FoxP3$^+$CD127$^-$ regulatory T lymphocytes, and activated CD8$^+$ T lymphocyte numbers in MIC patients R7 (A), R11 (B), R12 (C), and R14 (D) are shown. Transitional B lymphocyte numbers were closely related to the numbers of activated CD8$^+$ lymphocytes.
